# Molecular fragmentation as a crucial step in the AI-based drug development pathway

**DOI:** 10.1038/s42004-024-01109-2

**Published:** 2024-02-01

**Authors:** Shao Jinsong, Jia Qifeng, Chen Xing, Yajie Hao, Li Wang

**Affiliations:** 1 Nantong University, School of Information Science and Technology, Nantong, China; 2 Nantong University, Research Center for Intelligence Information Technology, Nantong, China

**Keywords:** Structure-based drug design, Virtual screening, Computational chemistry

## Abstract

The AI-based small molecule drug discovery has become a significant trend at the intersection of computer science and life sciences. In the pursuit of novel compounds, fragment-based drug discovery has emerged as a novel approach. The Generative Pre-trained Transformers (GPT) model has showcased remarkable prowess across various domains, rooted in its pre-training and representation learning of fundamental linguistic units. Analogous to natural language, molecular encoding, as a form of chemical language, necessitates fragmentation aligned with specific chemical logic for accurate molecular encoding. This review provides a comprehensive overview of the current state of the art in molecular fragmentation. We systematically summarize the approaches and applications of various molecular fragmentation techniques, with special emphasis on the characteristics and scope of applicability of each technique, and discuss their applications. We also provide an outlook on the current development trends of molecular fragmentation techniques, including some potential research directions and challenges.

## Abstract (web summary)

Molecular fragmentation plays a pivotal role in medicinal chemistry and drug discovery. Here the authors summarize the methodologies for molecular fragmentation and their application in the AI-based fragment-based drug discovery.

## Introduction

Over the past fifty years, the application of AI in drug design has never ceased. Following the successful prediction of approximately 200 million protein structures from over a million species using the Artificial Intelligence (AI) prediction algorithm AlphaFold2^1^, as unveiled by DeepMind, the field of AI-driven small molecule drug discovery has become a major trend at the crossroads of computer science and life sciences. However, the extent to which this technology can bring about changes in drug development in the short term still depends on the computer’s ability to understand and represent chemical space. A thorough and rational fragmentation of compounds is a crucial step in the computer’s understanding of compounds. By fragmenting compounds and identifying important correlations between substructures, a solid foundation can be established for subsequent work.

In the pursuit of novel compounds, fragment-based drug discovery (FBDD) has emerged as a new method, gaining increasing traction in the pharmaceutical industry. FBDD is employed to reduce losses and provide leads for biological targets that are challenging for traditional drug discovery. FBDD facilitates the optimization of low molecular weight ligands (~150 Da) into potent molecules with drug-like properties. In contrast to high-throughput screening, fragment-based methods require screening fewer compounds. Although initial potency hits may be lower, these methods offer more efficient and productive optimization approaches, significantly expanding chemical space. The rising Generative Pre-trained Transformers (GPT) models have demonstrated robust application capabilities across various domains. GPT’s essence lies in the pre-training and representation learning of linguistic units (characters or words), which relies on segmenting longer sentences following linguistic logic. If compounds are perceived as a language, the concepts of linguistic units like characters and words can be replaced with molecular fragments possessing specific functionalities. In this context, molecular fragments serve as the linguistic units of compounds. The inspiration for innovative drug discovery methods stems from rethinking the research paradigm of Quantitative Structure-Activity Relationship (QSAR), which fundamentally explores the relationship between substructures and activity. The problem of molecular substructure delineation bears resemblance to the challenges of sentence fragmentation and translation in Natural Language Processing (NLP), thus presenting opportunities for mutual insights. Large-scale generative models hinge on precise representations of compounds. In earlier studies employing Transformers for compound representation, the fragmentation of drugs into fragments has facilitated the convenient utilization of Transformer models to extract semantic relationships between compound substructures, significantly enhancing the model’s understanding of compounds^2,3^. Hence, fragmentation becomes a necessary and efficient step toward advancing AI-driven drug discovery.

As a fundamental strategy in medicinal chemistry and drug discovery, fragmentation involves the systematic dissection of complex molecules into smaller fragments. This approach offers insights into the structural features and interactions critical for molecular recognition and binding to biological targets. By breaking down intricate compounds into simpler components, researchers gain a deeper understanding of the underlying principles governing ligand-receptor interactions. Fragmentation techniques play a pivotal role in lead compound identification, optimization, and the exploration of chemical space, ultimately contributing to the development of novel therapeutic agents. In this context, the study of fragmentation methodologies and their applications continues to illuminate new avenues for efficient drug design and development.

This review first summarizes 15 methods for molecular fragmentation. It introduces the logic of fragmentation from the perspectives of fragmentation-based methods, sequence-based methods, and structure-based methods, and also proposes potential fragmentation methods. Secondly, it discusses the applications of molecular fragmentation. Finally, it suggests a strategy for selecting molecular fragments based on specific application scenarios.

### Significance of fragmentation

FBDD, as a fragment-based drug discovery approach, has emerged as a potent tool in the pursuit of novel pharmaceutical agents. It has evolved as a compelling alternative to the traditional method of lead compound identification through high-throughput screening (HTS). Distinguished from HTS, FBDD demonstrates the capability to recognize even smaller chemical components. Leveraging the concept of fragmentation, FBDD facilitates the acquisition of molecular fragments, allowing them to interact with distinct segments of biological target molecules. The extraction and analysis of fragment-target interaction relationships stand as a pivotal focal point within the realm of FBDD research.

Compared to HTS, FBDD offers several advantages. The primary rationale behind fragment-based screening is that hits identified through this approach can explore a broader chemical space while screening a limited number of compounds. FBDD provides a better opportunity for generating lead compounds with standard drug-like properties. Moreover, due to the complexity of interactions involving ligands and amino acid residues in active sites, relying on the identification of compounds perfectly matching the binding site of the intended target is not viable. Conversely, molecular fragmentation is a simpler approach, characterized by fewer inevitable interactions. As a result, it enhances the probability of achieving targeted interactions within the binding site.

The continued prominence of GPT has sparked extensive discussions on how GPT influences drug development. Traditional NLP research primarily relied on expert-crafted grammar and rules. However, in recent years, the advent of Transformers and attention mechanisms (including multi-head attention) has enabled models to grasp diverse relationships among tokens within input sequences. This paradigm shift has profoundly revolutionized the field of sequence data processing, constituting a pivotal breakthrough in natural language text comprehension.

**Fig. 1:**
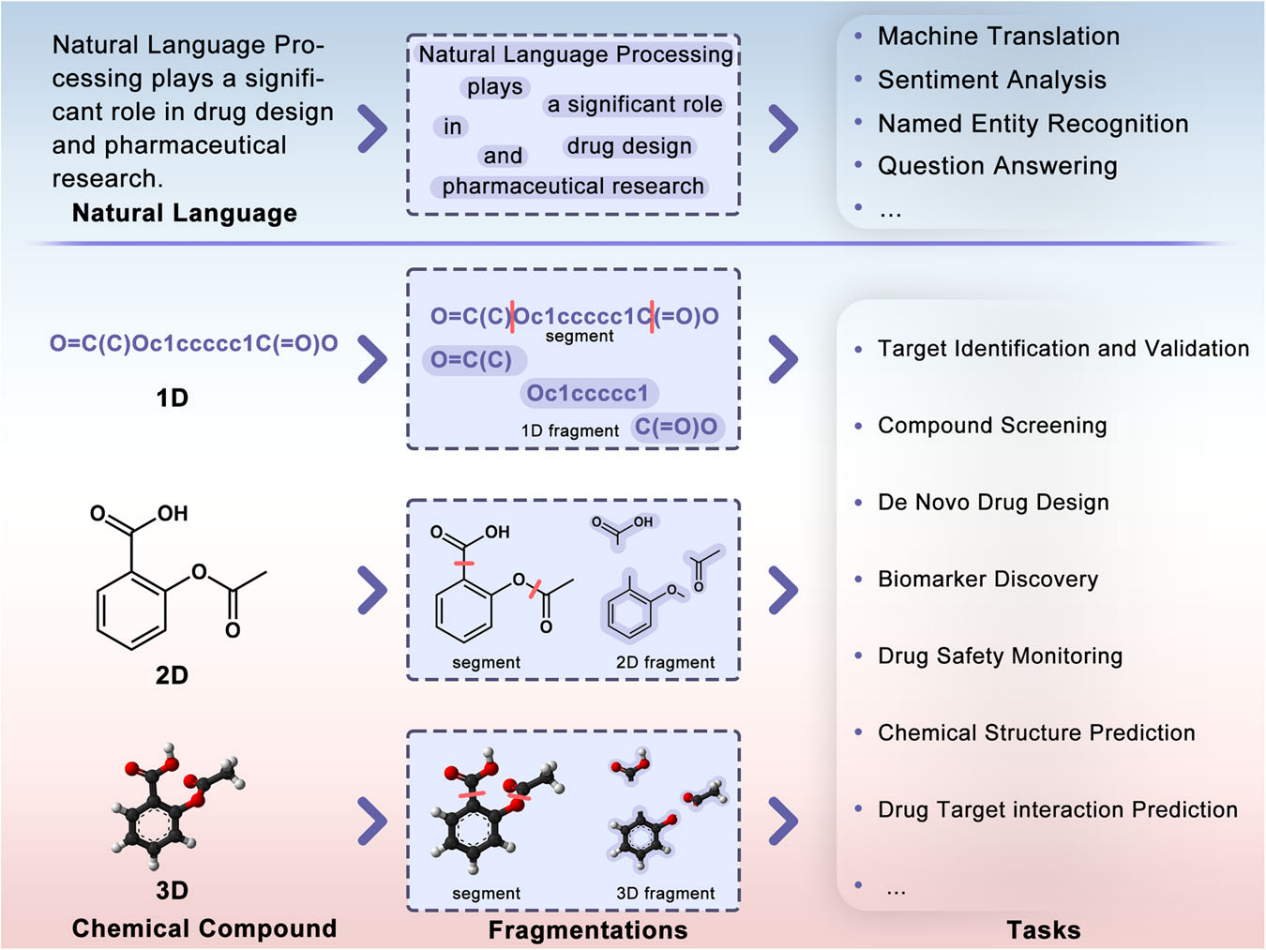
Molecular fragmentation technologies benefit downstream tasks. Effective molecular fragmentation technology can pay more attention to the local as well as global association and be more flexible in the application of downstream tasks, like the token extraction technology in NLP technology.

Chemical structures of molecules can be represented using linear encodings akin to the representation of natural language text. Similar to sentence segmentation in NLP, the delineation of molecular fragments bears a resemblance to this concept. Such words in a sentence can be harnessed for fragment-based drug discovery (Fig. 1). In a broader chemical space, molecular fragments composed of atoms and bonds can similarly construct more precise representation scenarios. At the same time, it gives more flexibility to the molecular representation process^4–6^.

However, the current challenge lies in determining how to derive fragment divisions from a vast pool of molecules that not only retains the integrity of chemical activity representation but also avoids excessive expansion that might result in an unwieldy and sparse fragment lexicon. Conventional approaches often rely on fragment libraries^7^, which are maintained over extended periods by numerous companies and pharmaceutical research teams. However, their high fragment copyright costs, limited fragment quantity, and uneven fragment distribution hinder their scalability for broader applications in universal drug discovery scenarios.

Due to the significant trend of AI molecular fragmentation and the limitations posed by the current usage of fragment libraries, employing large-scale nonexpertise-dependent fragmentation methods has become a crucial aspect of subsequent research. Thus, in this review, we comprehensively summarize the methods of fragmentation based on expert-driven approaches in recent years. Particularly, we expand on an array of non-expertise-dependent methods built upon these foundational approaches. Our review includes the elucidation and organization of fragmentation principles and implementation techniques, along with an extensive examination of issues stemming from method derivation. Some intricate methods are explained using figures to facilitate the reading and comprehension for beginners in the following sections.

This review summarizes and compiles the currently employed fragmentation methods. The executable and original code are organized and made available on a Github page for sharing, detailed at https://github.com/NTU-MedAI/MolFrag.

### Method of fragmentation

Molecular fragmentation is the process of dividing a large molecular compound into smaller molecular fragments. These smaller fragments can consist of individual functional groups or compound segments containing specific structural features. Molecular fragmentation finds widespread applications in fields such as computational chemistry, drug design, and chemical informatics. This work focuses on the systematic categorization and organization of methods related to molecular fragmentation. The categorization encompasses various aspects, including the mode of molecular fragmentation, whether specific structures (such as cyclic structures or double bonds) are disrupted, the retention of fragmentation information, and the incorporation of predetermined fragment libraries. These properties have been summarized and organized, as depicted in Table 1. As shown in Table 1, we list recommendations for the application tasks of current common fragmentation methods for reader evaluation.

**Table 1:** Summary of Fragmentation Methods.

Methods No.	Fragmentation method name	Dimension	Breaking of Cyclic Structures	Retention of break Bond Information	Inclusion of Predefined Fragment Libraries	Breaking Double Bonds	Task**
1	FCS^2^	1D	Yes	No	No	Yes	IP
2	Character Slicing (CS)^16^	1D	Yes	No	No	Yes	PP^49^
3	BPE^17^	1D	Yes	No	No	Yes	IP
4	SPE^18^	1D	No	No	No	No	MG
5	MMPs^21^	2D	No	Yes	Yes	No	IP, MG
6	RECAP^22^	2D	No	No	No	Yes	IP, MG
7	BRICS(BCS)^22^	2D	Yes	No	/*	Yes	IP
8	eMolFrag^23^	2D	No	No	No	Yes	IP
9	BPE_NLM^32^	1D	Yes	No	No	Yes	IP
10	MacFrag^26^	2D	Yes	Yes	Yes	Yes	/
11	FG splitting^27^	2D	No	No	No	Yes	IP, PP
12	FASMIFRA^29^	1D	No	No	No	Yes	MG
13	CReM^30^	2D	Yes	Yes	Yes	Yes	MG
14	UNIFAC^31^	2D	Yes	Yes	Yes	Yes	PP^50^
15	VOLT^33^	1D	Yes	No	No	Yes	IP

^*^The “/” symbol represents that it was not reported in the original text.

** IP represent the interaction prediction task; MG represent the molecular generation task; PP represent the properties prediction task.

#### Fragmentation based on existing fragment libraries

FBDD, based on fragment principles, holds pivotal significance for both the industrial and academic sectors as a vital strategy in drug design. Within the FBDD methodology, a selection of low molecular weight polar fragments/compounds is screened against specific targets. Common screening techniques involve biophysical methods, encompassing X-ray crystallography, nuclear magnetic resonance, differential scanning fluorimetry, isothermal titration calorimetry, surface plasmon resonance, and others^7^. One key factor in the success of FBDD is that the size of fragment-like compounds is smaller than that of drug-like compounds.

The latest advancements in computational tools and methods for fragment-based approaches have enhanced the identification of promising fragment hits. Typically, this approach begins with determining the structure of the target protein, followed by the preparation, docking, and hit confirmation of a virtual fragment library through molecular docking and molecular dynamics simulations. With the advancement of current technology, screening large libraries of fragments has become more feasible, resulting in higher hit rates. These fragments can subsequently be extended, merged, skipped, or linked to construct novel molecules.

In traditional FBDD practices, a majority of researchers opt to utilize existing fragment libraries for molecular fragmentation. Fragmentation through libraries is a prevalent chemical computation approach commonly employed in computer-aided drug design and molecular simulation research^7^. However, fragment-based fragmentation is a heuristic method that might not encompass all potential molecular fragments. Nonetheless, it often serves as a rapid screening or preprocessing technique. For intricate molecules, a combination of alternative fragmentation methods or manual intervention might be necessary to attain more accurate outcomes. Fragmentation through conventional chemical software packages like RDKit^8^ and Open Babel^9^ is widely applied in computational chemistry research.

Presently, a plethora of fragment libraries offer diverse fragment information. This work collates this information, as detailed in Table 2. This approach involves fragmenting molecules into segments containing specific substructures. Currently, predefined substructures (e.g., rings, heterocycles, and specific functional groups) can be defined and subsequently matched within molecules to facilitate fragmentation.

**Table 2:** Molecular fragment libraries.

Name	Link	Number of fragments
Life Chemicals General and Natural Product-Like	https://lifechemicals.com/screening-libraries/fragment-libraries	61,600
Aurora fine chemicals	https://aurorafinechemicals.com/targeted-library.html	8794
Otava general and natural product-like	https://otavachemicals.com/products/fragment-libraries	13,685
Enamine natural product-like	https://enamine.net/compound-libraries/fragment-libraries	220,000
Schrodinger Glide	https://www.schrodinger.com/products/glide	/
ACB Blocks	https://www.adbblocks.com	1280
ChemDiv	https://www.chemdiv.com	4283
IOTA	https://www.iotapharma.com	1500
TimTec	https://www.timtec.net	3200
Bioblocks	https://www.bioblocks.com	/
ZINC	https://zinc15.docking.org/	1,611,889

#### Sequence-Based Fragmentation

Before SMILES was widely popularized, there were many non-atomic ways to representation molecules, such as Wiswesser Line Notation (WLN)^10,11^, Hayward^12^ and Skolnik Notation^13^. These methods use tokens that represent functional groups, such as carboxyls or phenyls, as well as ring systems. SYBYL Line Notation (SLN)^14^ allows for macro atoms which specify multiple atoms in a substructure. The Hierarchical Editing Language for Macromolecules (HELM)^15^ represents complex biomolecules by declaring monomers and then connecting them in a polymer line notation.

Hakime et al. introduced the Character Slicing (CS) method in their work on DeepDTA^16^. The fragmentation products in CS do not correspond to traditional fragments. This approach employs the smallest constituent letters as minimal tokens, and during molecular representation, it directly employs fully connected layers to extract features from each token.

Rico Sennrich utilized the Byte-Pair Encoding (BPE)^17^ algorithm for molecular segmentation^17^. This method treats the compound SMILES as a sentence, determining the size of subwords. It then statistically counts the occurrence frequency of each consecutive byte pair and stores it as a codefile. Subsequently, words are split into character sequences, and the most frequently occurring byte pairs are merged based on the codefile. By iteratively merging, the subword vocabulary size reaches the set value, thereby completing the segmentation.

Frequent Consecutive Sub-sequence (FCS)^2^ shares a similar logic with BPE^17^, with the distinction that the Moltrans research team applied pre-fragmentation on a designated large dataset and then integrated it into the fragmentation of molecular substructures.

**Fig. 2:**
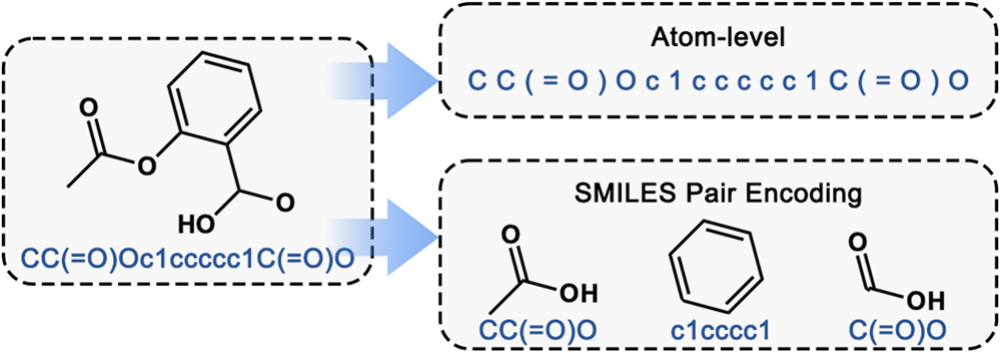
Fragmentation Logic of the SPE Method^18^. Compared to the atom-level tokenization model, SPE tokenizes SMILES for practical training of deep learning models based on the acquired vocabulary learned from extensive chemical datasets.

Sequential Piecewise Encoding (SPE) is a data-driven tokenization algorithm proposed by Xinhao Li et al. ^18^ SPE first learns vocabulary from extensive chemical datasets (e.g., ChEMBL^19^) regarding high-frequency SMILES substrings. Then, based on the acquired vocabulary, it tokenizes SMILES for practical training of deep learning models. In the process of molecular representation, the typical approach involves information extraction and correlation based on the atom level. However, SPE enhances widely used atom-level encodings by appending human-readable and chemically interpretable SMILES substrings as encodings for SMILES pairs (Fig. 2).

#### Structure-based fragmentation

Structure-based fragmentation is a method used in drug design and compound analysis, which involves breaking down compounds into molecular fragments to identify and optimize key features of drug molecules. Scaffold refers to the abstract representation of the core structure in a compound, possessing both invariance and variability. The methods for scaffold generation aim to identify and extract the shared core structures within a collection of compounds, facilitating improved analysis of structure-activity relationships and drug design. This means that Structure-Based Fragmentation can utilize the concept of Scaffold to identify and extract shared core structures in a collection of compounds, thereby playing a crucial role in drug design and structure-activity relationship analysis. In earlier work, Bemis and Murcko attempted to extract frameworks from molecules by analyzing the two-dimensional molecular structures, atom types, hybridization, and bond orders. This represented early work in structure-based fragmentation based on the efforts of researchers on scaffold, computational chemistry has accelerated, and we have found more structure-based fragmentation methods in recent work, which are derivatives of work on scaffold^20^.

Matched Molecular Pairs (MMPs) is a fragmentation algorithm introduced by Hussain et al. to simulate fragment linking scenarios^21^. Initially, the MMPs algorithm dissects each molecule using a dual cleavage approach, wherein non-functional groups and non-ring single bonds within each compound are doubly cleaved. This transformation converts the compound into a four-part structure: “fragment 1, linker, fragment 2, molecule” corresponding to two terminal fragments, a linker, and the original compound. All possible fragment molecule quadruples (FMQs) are enumerated. Subsequently, the FMQs undergo further screening based on the “rules of three” criterion, meaning that if any terminal fragment of an FMQ violates the “rules of three” criteria, the FMQ is discarded. The requirement for linker fragments is essentially to connect two proximate fragments using a linker that is as simple as possible. The remaining FMQs are subjected to additional filtration based on a Linker (Shortest Linker Bond Distance less than 15) and Synthetic Accessibility score (SAscore) using a filter. This ensures that terminal fragments possess reasonable synthetic feasibility (SAscore less than 5) and that the linker’s SAscore is lower than the sum of the fragments. This safeguards against the generation of highly complex linkers in the first generation.

The Retrosynthetic Combinatorial Analysis Procedure (RECAP) method, introduced by Jörg Degen et al., is the first approach within its category to apply 11 distinct rules for fragmenting active molecules to acquire active building blocks^22^. Certain common chemical reactions result in the formation of specific bonds, and it is these bonds that are cleaved. The presence of these 11 predefined bonds ensures that the resulting fragments are suitable for combinatorial chemistry. In this context, the concept of a fragment space is introduced. Unlike fragment libraries, such a space is not solely composed of a set of fragments but also a set of rules that dictate how fragments are reassembled by fusing their respective chemical motifs. A notable characteristic of this method is its capability to preserve the ring structure of compounds.

**Fig. 3:**
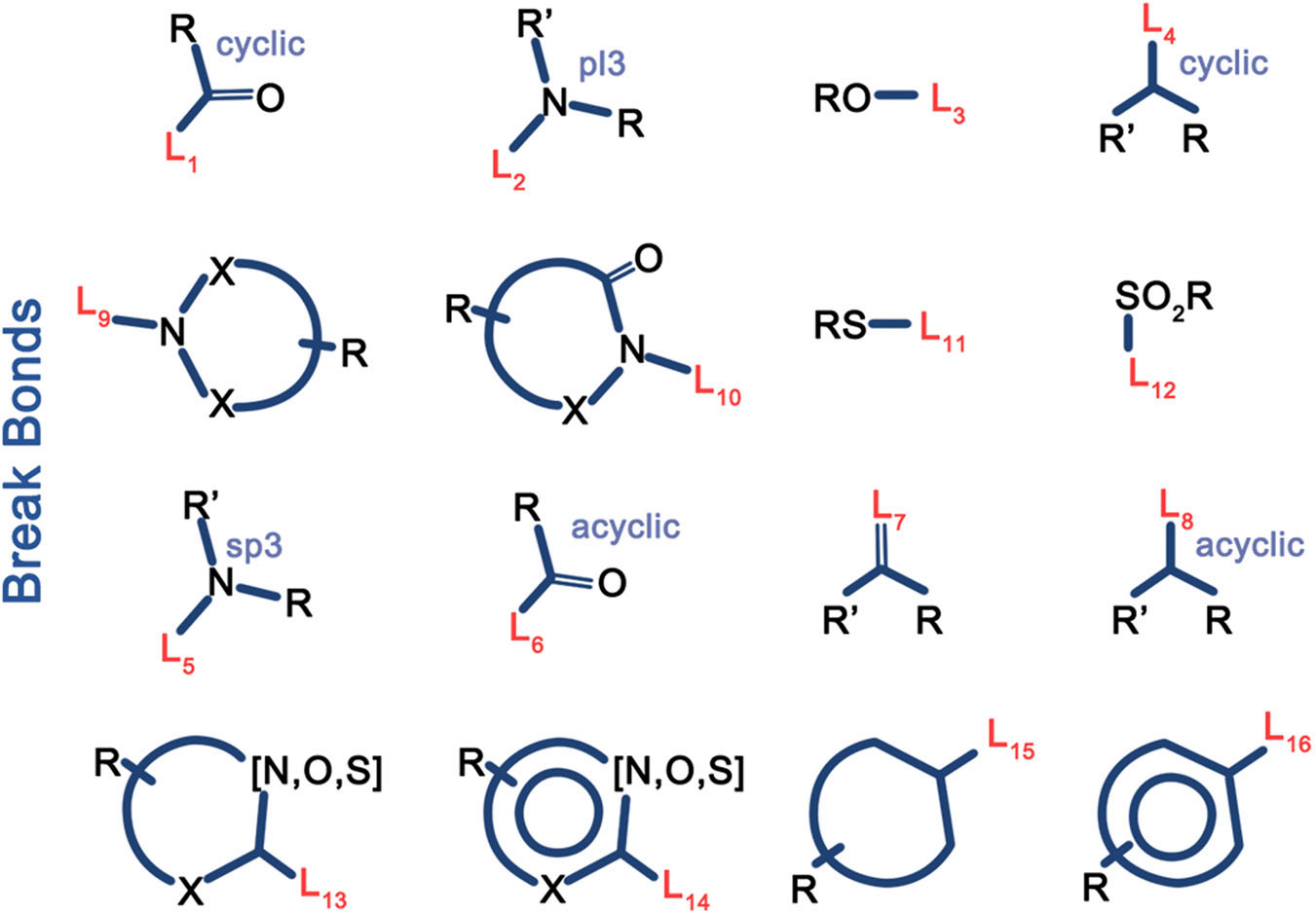
Fragment prototypes of the BRICS Method^22^. BRICS disrupts retro synthetically relevant chemical bonds within molecules to obtain various fragment information, corresponding to 16 distinct chemical rings.

Jörg Degen et al. simultaneously propose that compiling a corresponding fragment space by specifying a set of complementary rules for reassembly via respective chemical motifs is an effective way to partition compound fragments. Based on such rules, they develop a novel fragment-splitting approach called the breaking of retrosynthetically interesting chemical substructures (BRICS), which features 16 cleavage rules. This method disrupts retro synthetically relevant chemical bonds within molecules to obtain various fragment information, corresponding to 16 distinct chemical rings^22^ (Fig. 3). The approach extensively considers the chemical environment of each cleavage bond and its surrounding substructures.

Tairan Liu et al. proposed a compound fragmentation method named eMolFrag in their research work, where the functionality is primarily achieved by dividing molecules into two types of fragments: bricks and linkers, both of which belong to building blocks^23^. The eMolFrag approach divides molecules into fragment sets through two steps: (i) In eMolFrag, a set of molecules is initially decomposed using the BRICS algorithm implemented in RDKit. The molecules are broken down into fragments based on 16 chemical environments defined by the BRICS model, resulting in larger portions referred to as bricks. Brick fragments are molecular structures with at least four non-hydrogen atoms. Subsequently, bricks are removed from the molecules, and the remaining fragments are classified as linkers. Broken bonds are replaced by dummy atoms, which serve as placeholders for the atoms removed from specific bonds. Comprehensive information, including the types of atoms involved in the broken bonds, is stored on each brick to provide empirical connectivity patterns. Linkers possess distinct auxiliary connection information, indicating the maximum number of bonds annotated only at different positions on these fragments. (ii) Redundancy is eliminated to minimize the size of both bricks and linkers. If two fragments satisfy the condition of a Tanimoto coefficient (TC)^24^ of 1.0, determined through the topological constraints maximum common substructure calculation of the kcombu program^25^, the two fragments are considered equivalent and merged into the same fragment class.

**Fig. 4:**
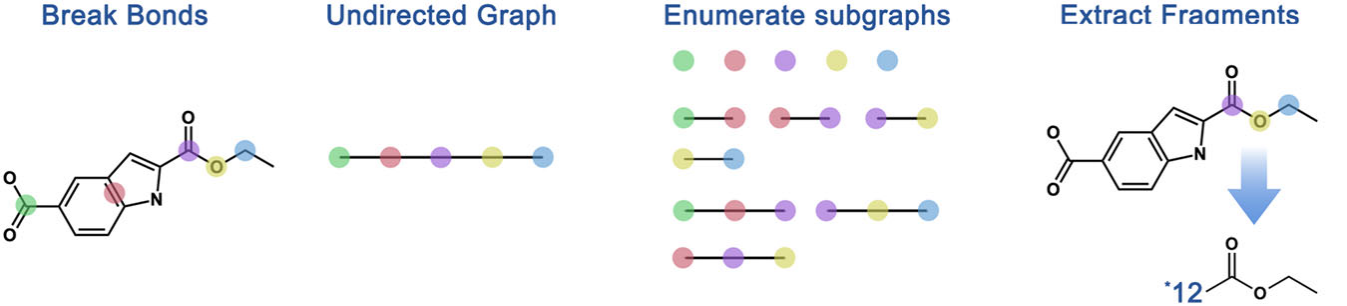
Fragmentation process of the Macfrag Method^23^. MacFrag fragmentation process involves breaking chemical bonds (recognize all cleavage bonds and cut he molecule), constructing undirected graph fragments (transform molecule into a graph with minimal building blocks as nodes), enumerating subgraphs (introduce a induced subgraph enumeration algorithm), and ultimately extracting fragments.

Yanyan Diao et al. introduce MacFrag in 2023, defines systematic ring-breaking rules and extends the BRICS method, thereby increasing the likelihood of obtaining novel molecular fragments^26^. The first step of MacFrag is to recognize all cleavage bonds and cut he molecule into the smallest building blocks. Then, atomic environments were defined using SMARTS strings, which were subsequently combined into bonds to be cleaved. A user-definable parameter *maxSR* is settled to determine whether the ring structure will remain intact by comparing the parameter and the ring structure containing the number of atoms. A very large value of *maxSR* means that the cyclic bonds will not be split, which is the same as the original BRICS version. The generated molecular fragments may encompass varying numbers of minimal building blocks, covering a broader fragment space to cater to diverse requirements in drug design tasks. The molecule is transformed into a graph with minimal building blocks as nodes, and an efficient induced subgraph enumeration algorithm called Simple is introduced, the fragments were extracted after removing extra atoms and bonds. The output fragments will be labeled with dummy symbols to specify the position of breaking bonds, and redundant fragments of the same molecule will be filtered. Figure 4 presents the complete MacFrag fragmentation process, which involves breaking chemical bonds, constructing undirected graph fragments, enumerating subgraphs, and ultimately extracting fragments.

**Fig. 5:**
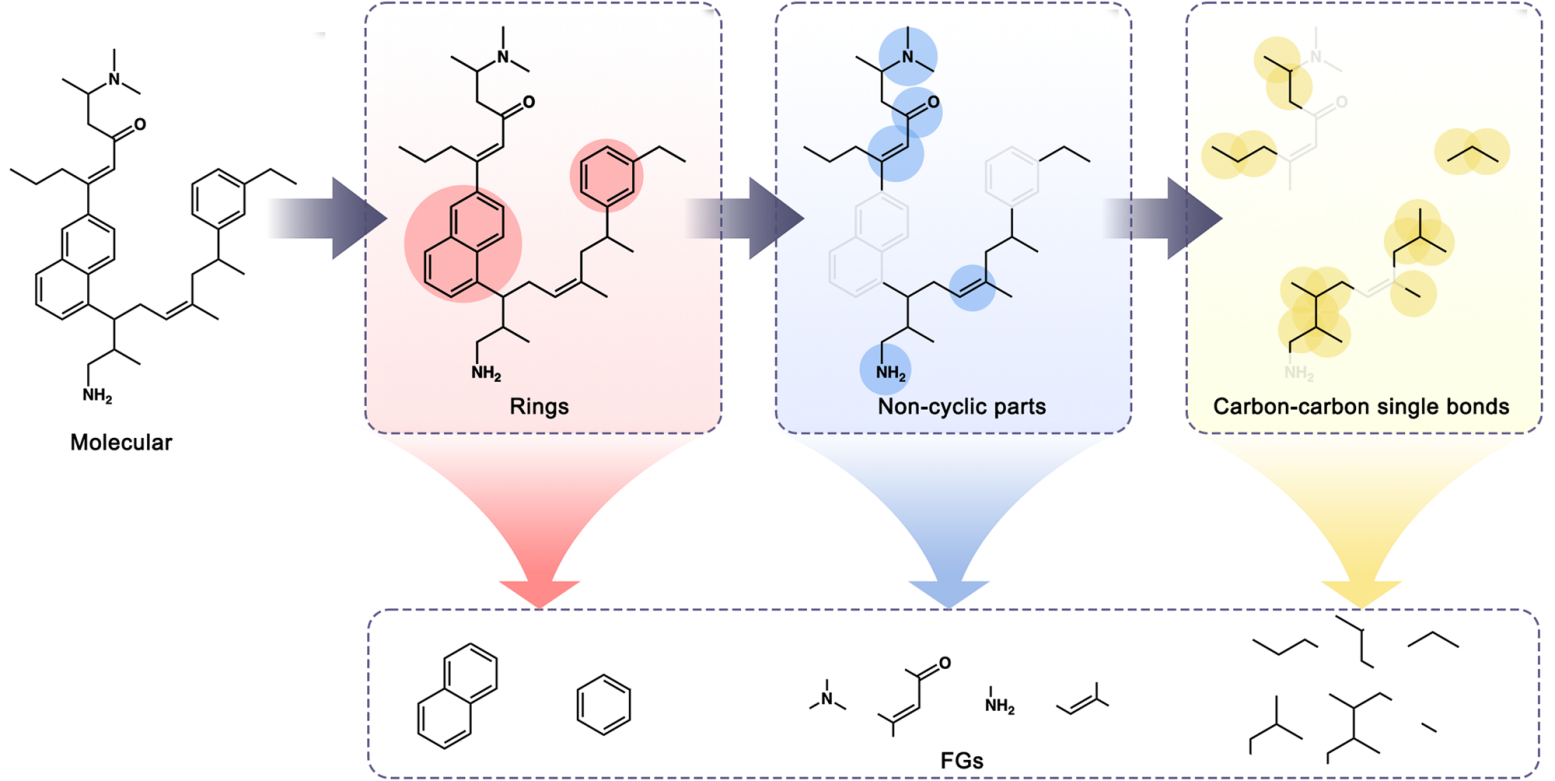
Strategies of ReLMole extracts FGs method. To transform molecules into Functional Group(FG) graphs of a large dataset, ReLMole is devised for the automatic extraction of FGs from atom-level graphs by defining three types of substructures (Rings, Non-cyclic parts and Carbon-carbon single bonds).

To transform molecules into Functional Group(FG) graphs of a large dataset, Zewei Jiden et al. devised an algorithm for the automatic extraction of FGs from atom-level graphs, termed FG splitting in their work ReLMole^27^. This method defines three types of substructures as building blocks for constructing FG-level graphs. Rings: For every ring structure within a molecule, it is designated as an FG. If two ring-form FGs share more than two atoms, implying the presence of bridge bonds between them, they are merged into a new FG. Non-ring FGs: In the non-ring portions of a molecule, functional atoms are first marked, encompassing heteroatoms, carbons in non-aromatic double or triple bonds, and aliphatic carbons bonded to two or more oxygen, nitrogen, or sulfur atoms. Subsequently, connected marked atoms and their adjacent carbons (i.e., those connected to unmarked carbons) are merged into FGs. Carbon-Carbon Single Bonds: Each Carbon-Carbon single bond not belonging to the previous two FG categories is also defined as an FG to achieve a complete partition on the atom-level graph. Based on the given definitions, a comprehensive list of substructures covering all compounds in the database can be generated, ensuring a unique and explicit partition. Since molecular graphs are partitioned into FGs, each FG is regarded as a node in the FG-level graph. If two FGs share any atoms, indicating adjacency in the atom-level graph, an edge is added to depict their connection (Fig. 5).

Xianbin Ye et al. proposed an alternative method known as Tree Decomposition^28^. When provided with a two-dimensional molecular graph G, the approach initiates by identifying all its simple cycles, with the graph’s edges not belonging to any cycles. If two simple cycles share two or more overlapping atoms, they are merged together as they form a distinctive structure known as bridged compounds. Each of these cycles or edges is treated as a cluster. Subsequently, a cluster graph is constructed by adding edges between all intersecting clusters. Finally, one of its spanning trees is selected as the connection tree for G. Due to cycle merging, any two clusters in the connection tree share at most two common atoms.

Francois Berenger and Koji Tsuda proposed another fragmentation logic based on molecular structures in FASMIFRA^29^. FASMIFRA performs molecular fragmentation by identifying bonds between heavy atoms which not in rings that can be cleaved. Meanwhile the bond must not be connected to a stereo center nor involved in cis-trans isomerism. The bonds that meet the above requirements will be randomly selected for cleavage. Excitingly, the authors propose a numerical calculation for how many fragments a molecule should be properly cleaved.

CReM is a structure-based generation method proposed by Pavel Polishchuk^30^. This method generates new molecular scaffold by mutating, growing and linking molecular fragments. They generate a database of interchangeable fragments in the following two steps: (i) structures of known compounds are exhaustively fragmented by cutting up to 4 non-cyclic single bonds between two heavy atoms using RDKit implementation of the matched molecular pairs algorithm. Hydrogens are cut separately. (ii) a context of a given radius is determined for attachment points of each fragment and encoded in a SMILES string.

Simon Muller developed a molecular fragmentation algorithm to deal with some challenges in automatic fragmentation, such as non-unique group assignment, incomplete group assignment and the composition of the fragmentation scheme^31^. To overcome the challenges, three features were implemented in his work: (i) Heuristic group prioritization. The patterns of the fragmentation scheme are sorted based on a set of heuristically determined descriptors. These descriptors can be, for example, the number of atoms describing the pattern, the number of bonds avail- able or the number of double bonds. (ii) Parent–child group prioritization. The complete fragmentation scheme is analyzed to find patterns that are contained within others. E.g. CH2 is contained in CONHCH2. Whenever searching for a specific pattern, if the group has such a parent pattern, the parent pattern is searched first. After that, the child pat- tern is searched. (iii) Adjacent group search. To avoid incomplete group assignments, whenever a part of the structure is already fragmented, the subsequent matches have to be adjacent to the groups already found.

#### Potential fragmentation methods

At present, most of the fragmentation methods are based on the improvement or combination of the existing methods. These methods share the same advantages as well as the same disadvantages. Cross-domain segmentation (e.g., NLP) may be an important potential way to solve the problem for many possible use cases. We list a few possible fragmentation methods, some of which have been validated in molecular fragmentation work but have not been published.

The BPE_NLM method is an extension of the BPE_NLM model proposed by Rafael-Michael Karampatsis et al., applied in the field of molecular fragmentation^32^. The approach of the BPE_NLM model utilizes BPE-selected subword units to predict tokens. This method introduces a caching mechanism to leverage source code locality while also adapting dynamically to new projects, such as molecular fragmentation tasks.

Vocabulary Learning via Optimal Transpot(VOLT) interprets molecular fragments as an exploration of tokenization, aiming to represent the optimal transport problem by finding an optimal token dictionary of appropriate size^33^. VOLT ranks all candidate tokens based on the pre-generated token frequencies. For simplicity, VOLT often adopts tokens generated by BPE (e.g., BPE-100k) as candidates. All tokens with probabilities are employed for initialization in the optimal transport algorithm, and at each time step, the vocabulary with the highest entropy can be obtained according to the transport matrix. Additionally, tokens with token frequencies less than 0.001 in the distributed character are removed. By exhaustively considering all time steps, a vocabulary satisfying the specified exponential search space is selected as the final vocabulary. The merged tokens in the vocabulary will combine two consecutive tokens into one token until no more tokens can be merged. Tokens outside the vocabulary are split into smaller tokens.

### Applications of fragmentation techniques

Building a high-quality library of molecular fragments is crucial for the successful fragment-based drug discovery. In the process of drug development, traditional computer-aided drug design methods often involve directly obtaining complete information about drug molecules for computer representation. Through the encoding and training process of input data, a relatively reliable functional application model can be obtained^34–39^. FBDD, namely Fragment-Based Drug Discovery, is a drug discovery method that differs from traditional high-throughput screening methods. Here are some advantages of the FBDD method:

(i) High Sensitivity^4^: FBDD is more sensitive, capable of detecting small and stable molecular fragments that may be overlooked by other methods. This increases the opportunity to discover new drug-candidate compounds.
(ii) Smaller Compound Libraries^40^: Compared to traditional large compound libraries, FBDD typically uses smaller fragment libraries. This reduces the number of compounds to be tested, improving efficiency.
(iii) High-Quality Starting Points^41^: FBDD often utilizes small molecular fragments that are chemically simple and easy to synthesize. Such fragments are more easily optimized into drug molecules with high affinity and selectivity.
(iv) Increased Drug Efficiency^42,43^: Since FBDD focuses on small and stable molecular fragments, it is easier to optimize them, enhancing the efficiency and pharmacokinetic properties of drugs.
(v) Better Understanding of Binding Sites^44^: FBDD typically employs methods such as X-ray crystallography to elucidate the binding mode between proteins and molecular fragments, providing more detailed structural information. This aids in a better understanding of the interaction between drugs and targets.
(vi) Reduced Compound Consumables and Costs^44^: Due to the use of smaller compound fragments, the cost of synthesis and testing is relatively lower, contributing to the overall reduction in the cost of the drug discovery process.
(vii) Increased Diversity of Drug Compounds^45^: FBDD often identifies diverse molecular fragments, thereby increasing the diversity of the final drug molecules. This is beneficial for overcoming drug resistance and enhancing efficacy.

In summary, the advantages of the FBDD method lie in its higher sensitivity and efficiency, allowing the discovery of small molecular fragments that may be overlooked by other screening methods, thereby providing more possibilities for drug discovery. However, how to choose the appropriate molecular fragmentation method to fully demonstrate the advantages of FBDD in practical application scenarios is still one of the main problems that perplexes researchers. It should be noted that in this review, we have included two molecular segmentation methods that are not currently actually reported. These two approaches are NLP-based text segmentation methods applied to 1D molecular representation encoding (e.g., SMILES and SELFIES). These methods had some good results in the field of NLP^32,33^, at the same time, through the test at hand in the interaction prediction task also has good performance.

### Selection of molecular fragment

When the purpose of molecular fragmentation is to facilitate molecular representation, the selection of fragments after splitting becomes particularly crucial. The screening of the fragment library generated through fragmentation is a vital step to obtain high-quality fragments. While this selection may lead to the loss of some information, the chosen molecular fragments can efficiently capture the inherent chemical properties within the molecule.

In the context of FBDD, compounds are screened, typically with fewer heavy atoms compared to conventional high-throughput screening sets. Hit identification methods must adapt to the smaller fragment size, necessitating sensitive biophysical techniques or higher concentrations for biochemical assays^46^. Theoretically, a carefully curated fragment library can cover a much larger proportion of chemical space than a carefully curated HTS library, providing researchers with increased confidence when constructing molecules from the fragment library^47^.

One of the earliest methods to describe fragment chemical properties is the “rule of three” proposed several years ago^48^. Other useful attributes can be utilized for fragment library screening, including quantitative evaluations of fragment purity, properties, stability, and solubility. More specific analysis criteria, such as fragment aggregation propensity and protein binding affinity, can also contribute to the selection process. Ultimately, the careful selection of fragments ensures that the chosen components effectively represent the chemical characteristics of the original molecules, enhancing the success of subsequent drug discovery processes and computational analyses.

When constructing a filtered molecular fragment library, the first consideration is the number of compounds to include in the fragmentation library; this is to some extent driven by the detection techniques used for fragment screening. High-throughput techniques, such as High Concentration Screening (HCS), often utilize biochemical assays and are generally less sensitive compared to low-throughput biophysical techniques. Generally, less sensitive techniques require more efficient fragments, which might be more complex compound fragments, implying the need for a larger fragment library.

Simultaneously, as ligand complexity increases, the probability of any fragment being hit decreases exponentially. A more common scenario is that fragment libraries are screened using sensitive biophysical techniques, where the fragment library usually needs to comprise several thousand compounds with molecular weights between 140 and 230 Da. Therefore, it is necessary to ensure that the library:

(i) Sampling relevant chemical space by incorporating key pharmacophores that drive fragment binding.
(ii) Incorporating an appropriate distribution and balance of fragments with adequate complexity and different shapes (refer to the section on 3d metrics). Overly complex fragments can reduce the hit rate as functionalities may interfere with binding; due to the entropy cost of constraints, a higher hit rate flexibility leads to lower intermolecular pharmacophore affinity.
(iii) Encompassing diversity of comprehensive accessible growth vectors to enable efficient optimization of fragment hits into lead compounds. Avoiding clusters associated with known and high-reactive, solution-aggregating, or persistently false-positive data^47^.

Small fragments often generate hits with low affinity and specificity, but these fragments can extract features that are involved in interactions with proteins. Low specificity has two consequences: first, fragments might bind to a variety of proteins; second, fragments could bind to a single protein in multiple ways. In the first sense, low specificity can enhance the hit rate in fragment screening, as specificity can be introduced later in fragment optimization. The ability of fragments to bind to proteins in multiple ways might hinder optimization strategies that assume consistent binding poses for establishing structure-activity relationships. However, fragments with multiple binding modes can still be valuable for druggability studies. They can be integrated into a comprehensive program to experimentally and computationally probe proteins with very small compounds, such as water and organic solvents, which bind to proteins containing a small number of non-hydrogen atoms and form clusters consistent with known inhibitor positions. Furthermore, the extension and relative positions of these clusters carry important information about the druggability of the protein.

## Outlook

In the future work, some new directions or outstanding problems are listed here. The first direction is expanding molecular fragmentation methods. Methods based on energy-based fragmentation can avoid cutting high-energy chemical bonds that are not easily broken, thereby preserving functional group fragments in chemical reactions. The second direction is preserving fragmentation information. After molecular fragmentation, in addition to retaining the structural information, it should also include information such as the position, energy, and the correlation of the fragments in the original molecule. The final direction is evaluating the quality of molecular fragmentation. In addition to relying on downstream task performance evaluations, it is necessary to establish a system for assessing the quality of molecular fragmentation directly from the fragment self. This ensures fairness in the evaluation of molecular fragmentation quality.
